# Concluding remarks

**DOI:** 10.1080/09629359791640

**Published:** 1997-11

**Authors:** Ragnar Rylander

**Affiliations:** 1 Department of Environmental Medicine University of Gothenburg Medicinaregatan 16 Gothenburg S-413 90 Sweden


Mediators of Inflammation, **6,** 289 (1997)

### Concluding remarks

**Ragnar Rylander**

Department of Environmental Medicine, University of Gothenburg, Medicinaregatan 16, S-413 90 Gothenburg, Sweden

The success of any scientific meeting is closely related to the number of new ideas or collaborative projects that are generated. In the workshop on Aspects of glucan toxicity, a number of important areas emerged in which there was agreement on further research and in which participants at the workshop will collaborate. These are outlined below.

It was accepted that there is a need to compare the two analytical methods now available: the Limulus lysate-related Fungitec G test [1] and a new immunological technique using antibodies [2]. From a theoretical point of view, there could be discrepancies between these techniques and knowledge of any possible discrepancies is essential for the definition of methodological requirements.

It was agreed that several different forms of (1→3)-β-D-glucan will be assayed in the first phase of a comparative study. These are schizophyllan, zymosan, grifolan, barley glucan and curdlan. The different preparations will be aerosolized and duplicate filter samples will be taken of the aerosol. Analyses will be made using the two methods.

In the second phase of this study, various environmental dust samples (cotton, swine confinement, waste handling) will be analyzed using the two methods, including the inhibition of (1→3)-β-D-glucan using glucanase.

In relation to different forms of (1→3)-β-D-glucan and environmental sampling, it was clear that water extracts from a number of different fungal microbes show essentially the same reactivity with the Fungitec G test. This might facilitate the analysis of environmental samples, where some basic form could be the most common form of (1→3)-β-D-glucan, as well as the clinical diagnosis of fungal infection. Knowledge about environmental (1→3)-β-D-glucan would also make it possible to use an optimal reference for the Fungitec G test. Differences in the basic structures and water solubility are already known to relate to differences in effect activity [3].

Data on important cellular activation processes, particularly regarding macrophages and neutrophil leukocytes, were presented at the Workshop. These data had been obtained from animal models using *in vivo* and *in vitro* techniques. Other data from inhalation studies failed to demonstrate an inflammagenic activity of the different forms of (1→3)-β-D-glucan when assayed in their natural form. However, both models indicated that (1→3)-β-D-glucans act in an additive or even synergistic fashion with bacterial endotoxin. Further work is needed on a larger range of (1→3)-β-D-glucans to exclude or confirm the presence of an inflammation-inducing property after inhalation. This is particularly important to an understanding of acute and chronic effects after environmental exposure. A collaborative project using grifolan was established.

The question of how (1→3)-β-D-glucan disappears from the environment was raised. Glucanases are produced by both bacteria and fungi and are thought to be important in an ecosystem. There is only limited knowledge about the effects on (1→3)-β-D-glucan of ultraviolet radiation, sunlight or chemical substances normally present in the air such as ozone. Better knowledge of this area could be useful in treating buildings contaminated with molds.

Data demonstrated during the workshop suggest that (1→3)-β-D-glucan could be an important environmental agent related to human disease. In particular, further work is needed on airborne (1→3)-β-D-glucan originating from mold growth indoors; both experimental and epidemiological studies are needed before a basis for control purposes can be established. The workshop provided an excellent basis for this work.

### References

1. Tamura H, Arimoto Y, Tanaka S, Yoshida S, Obayashi T, Kawai T: Automated kinetic assay for endotoxin and (1→3)-β-D-glucan in human blood. *Clin Chim Acta* 1994, **226**:109–112.
2. Douwes J, Doekes G, Montijn R, Heederik D, Brunekreef B: Measurement of (1→3)-β-D-glucans in occupational and home environments with an inhibition enzyme immunoassay. *Appl Environ Microbiol* 1996, **62**:3176–3182.
3. Ohno N, Miura NN, Chiba N, Adachi Y, Yadomae T: Comparison of the immunopharmacological activities of triple and single-helical schizophyllan in mice. *Biol Pharm Bull* 1995, **18**:1242–1247.

© 1997 Rapid Science Publishers

